# Bacterial Meningitis in Patients using Immunosuppressive Medication: a Population-based Prospective Nationwide Study

**DOI:** 10.1007/s11481-016-9705-6

**Published:** 2016-09-09

**Authors:** Kiril E. B. van Veen, Matthijs C. Brouwer, Arie van der Ende, Diederik van de Beek

**Affiliations:** 1 Department of Neurology, Center of Infection and Immunity Amsterdam (CINIMA), Academic Medical Center, University of Amsterdam, PO Box 22660, 1100DD Amsterdam, the Netherlands; 2 Department of Neurology, Medical Center Haaglanden, The Hague, the Netherlands; 3 Department of Medical Microbiology and The Netherlands Reference Laboratory for Bacterial Meningitis, Academic Medical Center, University of Amsterdam, Amsterdam, the Netherlands

**Keywords:** Corticosteroids, Bacterial meningitis, *Listeria monocytogenes*, Immunosuppressive medication, *Streptococcus pneumoniae*

## Abstract

We studied occurrence, presentation, disease course, effect of adjunctive dexamethasone, and prognosis of bacterial meningitis in patients using immunosuppressive medication. Patients were selected from our nationwide, prospective cohort on community-acquired bacterial meningitis performed from March 1, 2006 through October 31, 2014. Eighty-seven of 1447 episodes (6 %) of bacterial meningitis occurred in patients using immunosuppressive medication, and consisted of corticosteroids in 82 %. Patients with bacterial meningitis using immunosuppressive medication were less likely to present with headache (*P* = 0.02) or neck stiffness (*P* = 0.005), as compared those not on immunosuppressive medication. In 46 % of episodes CSF leukocyte count was below 1000/mm^3^. CSF cultures revealed *S. pneumoniae* in 41 % and *L. monocytogenes* in 40 % of episodes. Outcome was unfavorable in 39 of 87 episodes (45 %) and death occurred in 22 of 87 episodes (25 %). Adjunctive dexamethasone was administered in 52 of 87 (60 %) episodes, and mortality tended to be lower in those on adjunctive dexamethasone therapy as compared to those without dexamethasone therapy (10 of 52 [19 %] vs 12 of 35 [34 %], *P* = 0.14). We conclude that bacterial meningitis in patients using immunosuppressive medication is likely to present with atypical clinical and laboratory features, and is often caused by atypical bacteria, mainly *L. monocytogenes*. Adjunctive dexamethasone is widely prescribed in these patients and was not associated with harm in this study.

## Introduction

Community-acquired bacterial meningitis is a severe infectious disease with high morbidity and mortality rates (Brouwer et al. 2010), and is most commonly caused by *Streptococcus pneumoniae* (van de Beek et al. 2004b). Acquired immunodeficiency previously has been associated with an increased risk of bacterial meningitis (Adriani et al. 2013; van Veen et al. 2016; Weisfelt et al. 2010). One of the most common acquired conditions causing immunodeficiency is the use of immunosuppressive medication, including corticosteroids. The proposed mechanisms by which corticosteroids cause immunosuppression are decreased production, function and migration of inflammatory cells and decreased antibody production (Fardet et al. 2007). A meta-analysis showed an increased risk of systemic infections for patients using corticosteroids compared to patients not using corticosteroids, with a relative risk of 1.6 (95 % confidence interval [CI] 1.3–1.9), although it remains unclear whether corticosteroids or the underlying disease increased the risk of infection (Stuck et al. 1989). The use of immunosuppressive medication has been reported to be an important predisposing factor for infections with *Listeria monocytogenes* (Yildiz et al. 2007). Glucocorticoids can also mask the symptoms of infection and therefore delay treatment because patients present at an advanced stage of disease (Fardet et al. 2007).

Little is known about the clinical course of bacterial meningitis in patients using immunosuppressive medication.

Randomized clinical trials have evaluated the efficacy of adjunctive corticosteroid therapy in community-acquired bacterial meningitis (Brouwer et al. 2015; de Gans and van de Beek 2002; van de Beek et al. 2004a; van de Beek et al. 2010). As a result, adjunctive dexamethasone has been incorporated in treatment guidelines for bacterial meningitis (Tunkel et al. 2004; van de Beek et al. 2012; van de Beek et al. 2006). However, patients on immunosuppressive medication were excluded from the clinical trial and guidelines generally discourage the use of adjunctive dexamethasone in patients using immunosuppressive medication (Tunkel et al. 2004; van de Beek et al. 2012; van de Beek et al. 2006).

We studied clinical features and outcome of community-acquired bacterial meningitis in adults using immunosuppressive medication identified in a nationwide prospective cohort study, and evaluated the effect of adjunctive dexamethasone in this particular subgroup of patients.

## Methods

We conducted a nationwide, prospective cohort study on community-acquired bacterial meningitis. Methods have been described in detail previously (Bijlsma et al. 2016). From this cohort we selected all patients who were reported to use immunosuppressive medication. Immunosuppressive medication were classified in four main groups: glucocorticoids, small molecule drugs (e.g. cyclosporine, tacrolimus, everolimus, mycophenolate mofetil, azathioprine), protein drugs (e.g. horse or rabbit antithymocyte globulin, rituximab) and cytostatic drugs (Halloran 2004). Between March 2006 and October 2014, patients with bacterial meningitis over 16 years old were included. Bacterial meningitis was defined as a positive cerebrospinal fluid (CSF) culture or as a positive blood culture with a relevant pathogen, positive PCR or antigen test in cerebrospinal fluid, in combination with at least one CSF finding predictive of bacterial meningitis consisting of a CSF leukocyte count >2000 cells/mm^3^, polymorphonuclear leukocyte count >1180 cells/mm^3^, glucose level < 1.9 mmol/L, protein level > 2gL, or CSF/blood glucose ratio < 0.23 (Spanos et al. 1989).

Patients with a neurosurgical device, neurosurgical operation or procedure and patients with neurotrauma within one month of the onset of meningitis were excluded from the cohort. Data on patient history, symptoms and signs on admission, laboratory findings, radiologic examination, treatment, and outcome were prospectively collected by means of a case record form (CRF). Dosage of immunosuppressive medication was collected retrospectively from the discharge letters. For corticosteroids the dosage was calculated as the daily prednisolone equivalent dose. To this end dosage of hydrocortisone was divided by 4 and dosage of dexamethasone was multiplied by 6.7. All patients underwent neurologic examination at hospital discharge, and outcome was graded using the Glasgow Outcome Scale (GOS). A favorable outcome was defined as a score of 5, and an unfavorable outcome was defined as a score of 1 to 4.

The study was approved by the ethical committee of the Academic Medical Center.

Statistical analyses were performed with the use of SPSS statistical software, version 22 (SPSS Inc.). For numerical and ordinal data the student t-test or Mann-Whitney U test were used. For categorical data the Fisher exact test was used. Logistic regression was used to examine the association between potential predictors and the likelihood of an unfavorable outcome and of death. Odds ratios (OR) and 95 % confidence intervals were used to quantify the strength of these associations. Possible predictors of an unfavorable outcome and death were chosen on the basis of previous research (Koopmans et al. 2013; van de Beek et al. 2004b). All tests were 2-tailed, and *p* < 0.05 was considered significant.

## Results

### Patients and Immunosuppressive Medication

A total of 1447 episodes of bacterial meningitis were included in the cohort between March 2006 and October 2014. Medication on admission was known for 1435 patients (99 %). In 87 of these episodes (6 %) in 86 patients, patients were reported to use immunosuppressive medication.

The immunosuppressive treatment consisted of glucocorticoids in 71 episodes and was used as monotherapy in 45 episodes, and in combination with other immunosuppressive treatment in 26 episodes (Table 1). Auto-immune diseases (e.g. rheumatoid arthritis, ulcerative colitis) in 38 of 87 episodes (44 %) and cancer in 21 of 87 episodes (24 %) were the most frequent indications for immunosuppressive therapy. For corticosteroids, the median daily dosage was 20 mg prednisone (interquartile range [IQR] 7–30 mg). The median age of patients using immunosuppressive medication at the time of the episode of bacterial meningitis was 65 years (range 19–91 years; Table 2). Other co-existing conditions increasing the risk of meningitis were diabetes mellitus (in 16 episodes), alcoholism (in six episodes), and splenectomy (in four episodes). Distant foci of infection were identified in 25 of 82 episodes (31 %). One patient had a recurrence of bacterial meningitis during the study period and had a medical history of kidney transplantation.

**Table 1:** Groups of immunosuppressive agents and categories of indication

Immunosuppressive medication	n/N (%)	Indication	n/N (%)
Glucocorticoids	45/87 (52)	Auto-immune disease	38/87 (44)
Glucocorticoids + small molecule drugs	13/87 (15)	Rheumatoid arthritis	12/38 (32)
Glucocorticoids + cytostatic drugs	9/87 (10)	Ulcerative colitis	7/38 (18)
Glucocorticoids + protein drugs	3/87 (3)	Polymyalgia rheumatica	5/38 (13)
Glucocorticoids + SMD + PD	1/87 (1)	Auto-immune hepatitis	3/38 (8)
Cytostatic drugs	9/87 (10)	Systemic lupus erythematosus	3/38 (8)
Cytostatic drugs + protein drugs	1/87 (1)	Other auto-immune disease	8/38 (21)
Small molecule drugs	6/87 (7)	Cancer	21/87 (24)
Median dosage prednisone in mg^a^	20 (7–30)^b^	Renal transplantation	6/87 (7)
		Other	11/87 (13)
		Unknown	4/87 (5)

*PD* protein drugs, *SMD* small molecule drugs

^a^Known in 46 ﻿of 71 patients

^b^Interquartile range

**Table 2:** Comparison between patients with and without immunosuppressive medication^a^

Characteristic	immunosuppressive medication +	immunosuppressive medication –	P-value
Age (years)	65 (19–91)	61 (17–94)	0.002
Female	30/87 (34)	674/1348 (50)	0.006
Symptoms and signs on admission			
Headache	56/78 (72)	962/1161 (83)	0.02
Neck stiffness	51/85 (60)	945/1261 (75)	0.005
Triad of fever, neck stiffness, and change in mental status	31/86 (36)	543/1283 (42)	0.26
Absence of fever, neck stiffness, and change in mental status	7/87 (8)	43/1334 (3)	0.03
Predisposing factors^b^	23/87 (26)	266/1348 (20)	0.13
Distant focus of infection	25/82 (30)	581/1289 (45)	0.01
Blood chemistry tests^c^			
Leukocyte count (×10^9^/L)	13.9 (0.1–45.6)	17.1 (0.1–99.8)	0.0001
C-reactive protein (mg/L)	163 (3–500)	192 (0–752)	0.28
Indexes of inflammation in CSF^d^			
Leukocyte count (cells/mm3)	1368 (5–46,500)	2560 (0–463,149)	0.003
Leukocyte count <1000 cells/mm^3^	39/85 (46)	401/1234 (32)	0.02
Protein (g/L)	3.00 (0.45–11.0)	4.00 (0.02–50.0)	P < 0.0005
CSF/blood glucose ratio	0.17 (0.0–0.89)	0.04 (0.0–1.67)	0.003
Causative organism			
*Streptococcus pneumoniae*	37/87 (43)	981/1348 (73)	P < 0.0001
*Listeria monocytogenes*	35/87 (40)	43/1348 (3)	*P* < 0.0001
*Haemophilus influenzae*	6/87 (7)	44/1348 (3)	0.12
*Neisseria meningitidis*	2/87 (2)	147/1348 (11)	0.006
Other^e^	7/87 (8)	133/1348 (10)	0.71
Outcome			
Unfavorable outcome	39/87 (45)	495/1348 (37)	0.14
Mortality	22/87 (25)	219/1348 (16)	0.04
Neurological sequelae	18/53 (34)	360/997 (36)	0.88

*CSF* cerebrospinal fluid

^a^Data are presented as n/N (%), or median (range)

^b^Other than immunosuppressive medication

^c^Leukocyte count was known in 86 and 1327 episodes and C-reactive protein in 85 and 1283

^d^CSF leukocyte count was known in 85 and 1234 episodes, CSF protein levels in 85 and 1275 episodes, and CSF blood to glucose ratio in 77 and 1249 episodes

^e^See Table 2 for details of causative organisms

### Clinical Features

Headache occurred in 56 of 78 episodes (72 %), neck stiffness in 51 of 85 episodes (60 %), fever in 64 of 86 episodes (74 %), and a change in mental status (defined by a GOS score below 14) in 61 of 87 of episodes (70 %). The classic triad of fever, neck stiffness, and a change in mental status was present in 31 of 86 of episodes (36 %). In 7 of 87 of episodes (8 %), fever, neck stiffness, and a change in mental status were all absent. Headache and neck stiffness were less often present in patients using immunosuppressive medication compared to patients not using immunosuppressive medication (respectively 56 of 78 [72 %] vs 961 of 1161 [83 %], *P* = 0.02 and 51 of 85 [60 %] vs 945 of 1261 [75 %], *P* = 0.005; Table 2).

A lumbar puncture was performed in all patients. Independent predictors of bacterial meningitis (Spanos et al. 1989) were present in 72 of 87 (83 %) patients. Median CSF leukocyte count was 1368 cells/mm^3^ (IQR 443–3166 cells/mm^3^). Patients using immunosuppressive medication had lower CSF leukocyte count as compared to bacterial meningitis patients not using immunosuppressive medication (1368 cells/mm^3^ vs 2560 cells/mm^3^, *P* = 0.003) and more often a leukocyte count <1000 cells/mm^3^ (39 of 85 [46 %] vs. 401 of 1234 [32 %], *P* = 0.02).

Blood chemistry tests showed patients using immunosuppressive medication often had no or only mildly elevated whole blood leukocyte counts (median 13.9 × 10^9^/L [IQR 9.1–20.0 × 10^9^/L]). In 30 of 86 of episodes (35 %) patients had blood leukocyte counts of <11.0 × 10^9^/L.

Neuroimaging (computed tomography) was performed on admission in 75 of 87 episodes (86 %) concerning patients using immunosuppressive medication, and showed abnormalities in 26 patients: brain abscess in two, sinus or mastoid opacification in nine, generalized brain edema in eight, a hypodense lesion presumed to be infarction in three, subdural effusion in two, and hydrocephalus in one. Both patients with brain abscess had listerial meningitis. Of the patients with a brain abscess one presented with aphasia and confusion while the other had no focal neurologic deficits.

CSF cultures revealed *S. pneumoniae* in 36 episodes (41 %), *L. monocytogenes* in 35 episodes (40 %), *Haemophilus influenzae* in six episodes (7 %), and *Neisseria meningitidis* in two episodes (2 %). One patient had a negative CSF culture, but had a positive antigen test for *S. pneumoniae* as causative organism. Other causative organisms were *Escherichia coli* in two episodes and *Nocardia farcinica*, *Streptococcus anginosus*, *Streptococcus mitis*, and *Pseudomonas aeruginosa* in one episode each. Pneumococcal and meningococcal meningitis occurred less often in patients using immunosuppressive medication compared to those who did not (37 of 87 [43 %] vs. 981 of 1348 [73 %], P = <0.0001 and 2 of 87 [2 %] vs. 147 of 1348 [11 %], *P* = 0.006), whilst listerial meningitis was encountered more frequently (35 of 87 [40 %] vs. 43 of 1348 [3 %], *P* < 0.0001). Inadequate initial antimicrobial therapy was started in seven of 86 patients (8 %) and consisted of failure to start amoxicillin in patients with *L. monocytogenes* meningitis in five patients.

### Outcome

Outcome was unfavorable in 39 of 87 episodes (45 %) of bacterial meningitis in patients using immunosuppressive medication and in 22 of 87 episodes (25 %) the patient died. The rate of unfavorable outcome was similar between patients with and without immunosuppressive medication (39 of 87 [45 %] vs. 495 of 1348 [37 %], *P* = 0.14) but mortality was higher in those using immunosuppressive medication (22 of 87 [25 %] vs. 219 of 1348 [16 %], *P* = 0.04). In a univariable analysis, the use of immunosuppressive medication was associated with death (OR 1.75; 95 % CI 1.05–2.89, *P* = 0.03). In a multivariable analysis adjusting for age (categories: < 40, 40–70, > 70 years), otitis or sinusitis, diabetes mellitus, neck stiffness, score on GOS, CSF leukocyte (<1000 cells/mm3), CSF protein (g/l), thrombocyte count (< 150 × 10^12^/L), C-reactive protein (mg/l) and *L. monocytogenes*, the use of immunosuppressive medication was no longer associated with death (OR 0.94, 95 % CI 0.46–1.93, *P* = 0.86).

Neurological sequelae occurred in 18 of 53 surviving patients (34 %) and consisted mostly of cognitive impairment in eight of 52 episodes (15 %) and cranial nerve palsies in eight of 53 episodes (15 %). The use of immunosuppressive medication did not influence the risk of sequelae (OR 1.09; 95 % CI 0.62–1.91; *P* = 0.76).

### Adjunctive Dexamethasone Therapy

In 52 of 87 episodes (60 %), patients were treated with adjunctive dexamethasone according to guideline recommendations (10 mg four times per day for 4 days, administered before or together with the antibiotics). In three episodes dexamethasone was started but discontinued because *L. monocytogenes* was identified as the pathogen. Baseline characteristics between those who did and did not receive dexamethasone were similar. Unfavorable outcome was observed in 21 of 52 episodes (40 %) of patients treated with adjunctive dexamethasone and in 18 of 35 episodes (51 %) not treated with adjunctive dexamethasone (*P* = 0.289). There was a trend towards protection against death for patients treated with dexamethasone (10 of 52 [19 %] vs 12 of 35 [34 %], *P* = 0.14). In an univariable analysis, for patients using immunosuppressive medication the OR of adjunctive dexamethasone treatment for death was 0.46 (95 % CI 0.17–1.22, *P* = 0.12). The effect size was not influenced by known predictors of death – age, score on GOS and *L. monocytogenes* as causative organism – in a multivariable analysis (OR 0.46; 95 % CI 0.16–1.31; *P* = 0.15). No adverse events related to the treatment with adjunctive dexamethasone were reported.

## Discussion

Patients with bacterial meningitis using immunosuppressive medication are less likely to present with typical clinical characteristics of meningitis, such as headache and neck stiffness, and have less marked CSF white cell counts. About half of patients had a CSF white cell count below 1000 per mm^3^; however, about 80 % had one of more independent CSF predictors of bacterial meningitis (Spanos et al. 1989). Cultures often reveal *L. monocytogenes* (40 %) or other atypical causative organisms, such as *Nocardia* or *Pseudomonas*. To prevent diagnostic and therapeutic delay, a low threshold should be kept for performing a lumbar puncture in patients using immunosuppressive medication, even those with a low clinical suspicion of bacterial meningitis. Adjunctive dexamethasone is widely prescribed for patients using immunosuppressive medication who are admitted with bacterial meningitis and is not associated with harm.

The association of *Listeria* infections with immunosuppressive medication, and glucocorticoids especially, has been well recognized by earlier studies (Hooper et al. 1982; Yildiz et al. 2007). The predilection for immunocompromised hosts may be explained by the intracellular life cycle of *L. monocytogenes* by which it avoids host bactericidal mechanisms. Resistance to infection is predominantly mediated by cellular immunity and their elimination requires T lymphocytes mediated cytotoxicity (Horta-Baas et al. 2013). Guidelines advise the use of a third generation cephalosporin combined with ampicillin or amoxicillin if *L. monocytogenes* is suspected (Chaudhuri et al. 2008; Tunkel et al. 2004). Based on the high frequency of *L. monocytogenes* as causative organism in patients using immunosuppressive medication identified in our cohort, empiric treatment for patients using immunosuppressive medication should be broad and include ampicillin or amoxicillin for listeria coverage in combination with an extended spectrum cephalosporin.

Adjunctive dexamethasone did not influence rates of unfavorable outcome. However, there was trend towards protection against death for patients treated with dexamethasone. This observed effect size was similar to that of the total population of bacterial meningitis patients and was not influenced by known other risk factors of mortality (de Gans and van de Beek 2002). Patients treated with adjunctive dexamethasone did not experience a higher rate of dexamethasone-related complications such as hyperglycemia requiring insulin, gastric bleeding, and herpes simplex infection. Randomized controlled trials on the effect of adjunctive dexamethasone in bacterial meningitis either do not report on the use of corticosteroids or immunosuppressive medication (de Gans and van de Beek 2002; Molyneux et al. 2002; Nguyen et al. 2007), or have glucocorticoids or immunosuppressive medication as an exclusion criterion (Peltola et al. 2007; Scarborough et al. 2007). With the number of patients included in this analysis we lack power to identify a significant association of dexamethasone, but we found a similar effect as was identified in previous studies. Based on these findings the use of adjunctive dexamethasone should be considered in patients using immunosuppressive medication.

Our study has limitations. The observational design of the study is sensitive to the introduction of confounding factors, which hinder the evaluation of dexamethasone effectiveness.

Other limitations of this study were that only patients with culture-proven meningitis were included in our cohort study. Not all patients with suspected bacterial meningitis may undergo a lumbar puncture, e.g. patients with coagulopathy due to sepsis or those with space-occupying lesions on cranial imaging. These patients were not included in our cohort. As the use of immunosuppressive medication is a risk factor for brain abscess, we could have missed cases of bacterial meningitis in patients use of immunosuppressive medication (Selby et al. 1997).

In conclusion, patients with bacterial meningitis using immunosuppressive medication are less likely to present with typical clinical characteristics of meningitis and have less marked CSF abnormalities. Cultures often reveal *L. monocytogenes* (40 %) or other atypical causative organisms. Adjunctive dexamethasone is widely prescribed for patients using immunosuppressive medication who are admitted with bacterial meningitis and was not found to be associated with harm, although studies in larger sample size are needed to validate these findings.
